# Qualitative study of the learning and studying process of resident physicians in China

**DOI:** 10.1186/s12909-022-03537-x

**Published:** 2022-06-15

**Authors:** Gui Chang Pan, Wei Zheng, Shih-Chieh Liao

**Affiliations:** 1 Department of Education of the Sixth Affiliated Hospital of Guangzhou Medical University/The People’s Hospital of Qingyuan, Guangzhou, China; 2 School of Medicine, China Medical University, 91, Shueh-Shih Road, Taichung, 404 Taiwan

**Keywords:** Residency training program, Clinical education, Qualitative study, Education ecosystem

## Abstract

**Background:**

Clinical medical education is essential in physician training. This study developed recommendations for medical residency course design on the basis of the perspectives of learners in China and how they interact with their environment. The central research topic was the professional development and learning process of residents, including the obstacles that hinder and factors that promote their learning, their views on existing teaching methods, interaction between teachers and medical teams, and suggestions for designing future residency training programs.

**Methods:**

This study had a qualitative research design. Interviews were conducted between July and October 2019 with 17 specialist residents and 12 assistant general practitioner residents from the department of education of the hospital. The participants were recruited from Qingyuan People’s Hospital in Guangdong Province, China. The interview outlines focused on the following four themes: clinical learning experiences and reflections on learning, experience of interaction with patients, experience of working with other medical personnel, and future learning directions.

**Results:**

To overcome challenges in clinical learning, the residents mainly learned from their teachers and focused specifically on their own experiences. Regarding teaching methods and designs in clinical medicine, the residents preferred large-group, small-group, and bedside teaching and reported that bedside teaching enables the resolution of clinical problems, initiates self-learning, and improves diagnostic thinking. They disliked teachers with low teaching motivation or who were reluctant to interact with them and favored teachers who had strong teaching skills and respect for their students.

**Conclusions:**

The residents suggested that clinical and active learning must be the main learning method for developing general medical competencies. Residency training must be conducted in an environment that facilitates residents’ learning and meaningful learning activities. The interdependent symbiotic relationships in the education ecosystem can serve as a reference for designing residency courses.

**Supplementary Information:**

The online version contains supplementary material available at 10.1186/s12909-022-03537-x.

## Introduction

Clinical medical education is a key part of the training of physicians. In medical education, a large gap exists between the application of theory and facts. Bedside teaching is a valuable teaching modality in medical education and is favored by medical students [1]. The apprenticeship model of medical education immerses students in a clinical setting, enabling them to learn by seeing and doing [2, 3].

As soon as medical students transition into residency, they are no longer students but physicians. Their interpersonal interactions include first-line engagement with patients and families instead of only face-to-face interactions with teachers. Residency training programs are implemented to improve students’ medical knowledge and clinical skills. Thus, resident physicians (or residents) learn through diverse instructional methods (e.g., group discussion) in addition to teaching, conference-room demonstrations, and informal chalk talk [4, 5].

Dewey asserted that experience is the product of the interaction between an organic body and the environment in which it is situated and that education involves the continual recall of and reflection on experience [6]. In certain environments, students have explicit or implicit cognitive affordances for learning, which stimulate human-perception-driven behavior and thus enable responses to the environment or engagement in learning [7, 8]. Educational ecology theory posits that learning and development occur in a multilevel cultural ecosystem, where human development represents the process of adaptation to the constantly changing environment [9]. Educators can integrate various ecological factors such as teacher-student relationship and student-student relationship to enhance the effectiveness of their teaching. Educational ecology theory originates from child development research [8]. An increasing number of higher education researchers are using the ecology of education to explore student learning processes and course designs [10] as well as medical education [11, 12].

In 2014, China implemented an education system involving dual residency [13] in which 5-year tertiary education is coupled with 3-year residency training or a 3-year master’s degree program in clinical medicine to train specialists for hospitals. Moreover, 3-year specialty education is coupled with 2-year assistant general practitioner (GP) training to train assistant GPs for basic-level health institutions.

Medical residents in China have now been presented with a revised training system that has a new curriculum design. The effectiveness of a curriculum depends not only on the content of the courses but also the learning environment, which has a major effect on students’ learning motivation. The purpose of this study was to examine whether the revised medical resident training program provides an effective and supportive learning environment for medical residents. Specifically, we investigated whether the pedagogical approaches were effective. Did instructors’ teaching style influence the residents’ learning outcomes? What challenges do residents encounter in the learning environment? Answering these questions and determining residents’ preferred teaching methods and possible learning challenges can assist program providers in making appropriate revisions to the program that enhance the effectiveness of medical resident educational training.

On the basis of interviews with medical residents, we explored how residents interacted with other medical personnel and with patients and their families during learning and studying processes. We also obtained their evaluations of different teaching methods and instructors. One study revealed that contextual change influences the activities, evaluations, and assessments of medical education curricula [12]. Therefore, this study examined whether sociocultural factors in the ecosystem of residency education influenced medical residents’ learning and studying processes. On the basis of the learning process of medical residents in China and discussion of the literature on various cultural environments, we compared the learning and studying processes of residents in different ecosystems. The research results can contribute to the future design and implementation of medical education curricula.

## Method

### Participants

Three residents were invited for a pilot study in May 2019. For the pilot study, we developed a preliminary version of the interview guide by conversing with residents and reviewing the related literature. Using the results of the pilot study, we revised the interview guide to create a formal interview outline to be applied in the main study.

Official interviews were conducted between July and October 2019. We publicized the research participant recruitment information on the social media (WeChat) of the residents of Qingyuan People’s Hospital. The information communicated the rights and duties of participants as well as the interview content and research purpose. All residents who volunteered to participate were automatically enrolled as participants, and participants were recruited until the data were saturated, after which no more participants were accepted.

No more interviews were held when the interview results of the 29th resident provided no new insights. Thus, 29 participants were recruited, comprising 17 specialist residents and 12 assistant GP residents. Of the 17 specialist residents, 7 had a master’s degree (4 women and 3 men), and 10 had a bachelor’s degree (4 women and 6 men). All the 12 assistant GP residents had a junior college degree (8 women and 4 men; see Table 1).

**Table 1:** The characteristics and numbers and of study participants

Group	Gender	Education
Specialist (17)	Female (8)	Master (4)
		Bachelor (4)
	Male (9)	Master (3)
		Bachelor (6)
Assistant GP (12)	Female (8)	Junior College (8)
	Male (4)	Junior College (4)

### Patient and public involvement

This study did not involve patients. In the hospital’s 2020 teaching review meeting, the results of this study were discussed with all residents. Moreover, the results of this study were used for the faculty development curriculum design. This study did not employ randomized controlled trials.

### Content of interviews

The interview outline comprised the following four main themes: clinical learning experience and reflection on learning, experience interacting with patients, experience working with other medical personnel, and future learning directions (see Additional file 1). To encourage participants to reflect on the questions, the interviewer discussed each interview question with the participants and encouraged them to ask questions to clarify the interview content during the interview.

### Research ethics and interview procedure

This study was reviewed and approved by the Institutional Review Board of Qingyuan People’s Hospital in Guangdong, China (IRB: QPH-IRB-A0141). Recruitment information was posted on the WeChat group of the residents of the hospital, and anyone who was interested in participating contacted the department of education of the hospital. Each interview lasted approximately 50–70 min and was held in a quiet, private meeting room in the Medical Education Development of Qingyuan People’s Hospital. The content of the interviews will not be shared with any third party.

SCL is the corresponding author of this paper and was the sole interviewer in this research. SCL does not work at Qingyuan People’s Hospital and had never met any of the research participants before their interview. SCL did not know any personal data of the participants. The participants were informed that the interview content would not influence their future academic scores and that they could therefore fully express their opinions without reservations.

The interviewer maintained a respectful attitude toward the participants and ensured that they were as comfortable as possible during the interview. The interviewer avoided guiding the responses of the participants to prevent causing information bias.

All the interviews were audio recorded with the participants’ permission. Verbatim transcripts were prepared and verified by the participants prior to further analysis.

### Analysis

A combination of inductive and deductive thematic analyses was employed to identify patterns of meaning across a qualitative data set [14]. The inductive analysis method was performed by drawing on residents’ experiences to identify patterns of themes and subthemes. The deductive analysis was conducted using educational and learning theories to identify patterns of themes and subthemes. In the analysis of this study, WZ, GCP, and SCL organized the transcripts of the interviews sentence by sentence, conceptually coding phrases to generate thematically meaningful contextual data.

GCP and WZ are attending physicians with 20 and 5 years of experience in medical education administration, respectively. SCL has a Ph.D. degree in educational psychology and has been specializing in the design and evaluation of medical education curricula for 20 years. GCP and WZ analyzed the interview data from the perspective of medical practitioners, and SCL analyzed the interview data from the perspective of educational psychology. The results of these two modes of data interpretation were compared and integrated to prevent the authors’ personal backgrounds and experiences from affecting the analysis and interpretation of the data.

The participants’ identities and the sequential order of the sentences spoken during the interview were assigned a code to enable us to quote and analyze the data. Responses provided in Mandarin have been translated into English, and each quotation has code that also includes the corresponding participant’s deidentified number (S#).

## Results

**Fig. 1:**
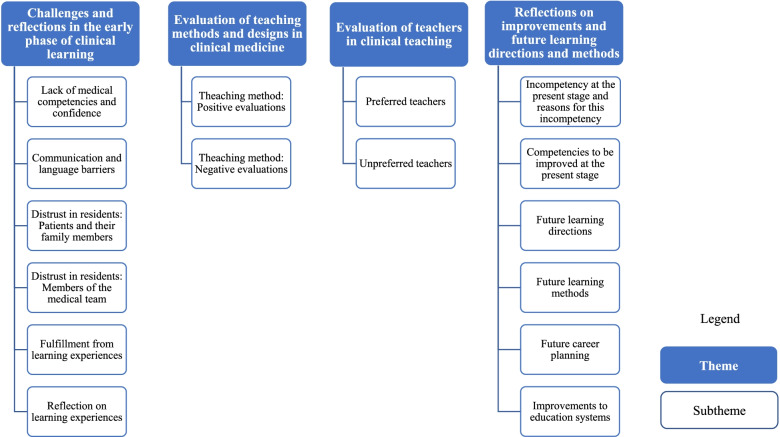
Themes and subthemes in the learning and studying processes

The challenges faced during clinical learning, evaluations of teaching methods and designs in clinical medicine, evaluations of teachers’ bedside teaching, and future learning directions and methods were determined. Example quotations relating to all themes and subthemes are listed in Table 2, and Fig. 1 presents these themes and subthemes in the order of the stage of the resident’ training.

**Table 2:** Themes related to learning processes in interviews with 29 residents

Themes and subthemes	Sample comments (student identifier)	No. of references^**a**^
**Challenges and reflections in the early phase of clinical learning**		38 (29)
Lack of medical competencies and confidence	I may have my own method of thinking when encountering common diseases. However, for complex cases, such as when patients have comorbidities, I’m inadequately equipped to treat and prescribe medicines to them (S6).	21 (18)
Communication and language barriers	When I first came here, because I barely interacted with [patients] and was completely inexperienced, whenever [patients] saw me, they read my name tag and asked if I was a resident. At first, they did not trust me…For example, in a task as simple as history taking, when I had just begun my residency, I used to stutter when asking a patient about their medical history. It did not go well (S23).	24 (19)
	Because I am not from Guangdong, I do not speak Cantonese…It was difficult to ask [the patients] precise questions (S28).	
Distrust in residents: Patients and their family members	[Patients] doubted us and asked us questions repeatedly, probably because [residents] look young. Sometimes, they asked us difficult questions that we were unable or not allowed to answer. We would then tell them that we must ask our superior. In such instances, they did not want to ask us any more questions but preferred to ask our teacher questions directly because they did not trust us (S26).	20 (17)
Distrust in residents: Members of the medical team	People do not trust younger doctors. Even nurses do not trust us (S21).	7 (6)
Fulfillment from learning experiences	[I] wanted to be a doctor to cure patients…We can diagnose and treat simple common diseases by ourselves. Sending patients home after they are cured is fulfilling (S12).	1 (1)
Reflection on learning experiences	[I] feel that there is still a lot to learn…Medicine is about accumulating experience…The greater the number and variety of cases you handle, the more experience you gain (S22).	18 (17)
**Evaluation of teaching methods and designs in clinical medicine**		
Teaching method: Positive evaluations		49 (29)
Large-group teaching	A broad range of knowledge is taught in large classroom settings (S27).	6 (5)
Small-group teaching	Because [teachers] use cases of actual patients, such as the cause of the onset of a patient’s disease, I believe that this approach not only helps us learn but also provides us with an in-depth look at clinical examples. I am receptive to this particular type of learning (S18).	14 (10)
Bedside teaching (physicians)	This patient is your patient. You can have face-to-face interactions with them. You can completely understand the disease from diagnosis to treatment. If you do not understand something, you can read texts and ask your teacher questions. In this manner, you obtain greater understanding of the disease (S8).	17 (10)
	In bedside teaching, the director leads all the doctors in the department around the ward and selects specific patients so they can explain difficult cases. The director asks us challenging questions that require discussion. The next step is diagnosis and treatment. The director encourages us to broaden our thinking. After completing a round, we return to the conference room and review the patients’ information. The director asks us questions individually and discusses cases with us, enabling us to develop our diagnostic thinking (S2).	
Bedside teaching (nonphysicians)	Patients sometimes believe that nurses are better than doctors. I believe that I can learn some communication skills from [nurses]… [Nurses] are more considerate than others. They pay more attention to patients’ problems than we do, and patients recognize that they care about them (S18).	10 (9)
	Pharmacists might know more about medications than we do. We can ask them for tips, interact with them, or question why their approach is preferable to ours. This process represents the process of learning (S29).	
Effective teaching methods in skills training and competitions	A clinical skills competition is organized every year…It is a way of rewarding us in our training program and makes us more eager [to acquire skills] (S12).	2 (2)
Teaching method: Negative evaluations		17 (10)
Large-group teaching	Some of the knowledge imparted is a bit too complex for me to understand (S3).	11 (8)
	Sometimes, teachers are overly focused on the data in a paper, which is not exactly useful for novices like us…Those who focus excessively on statistics or big data probably do not know us well yet (S12).	
Small-group teaching	A rule states that we must attend study class [small-group learning] every week. Some departments are very busy, and no one has the time to attend these classes… I believe that [small-class learning] is quite tiring (S16).	6 (3)
Bedside teaching	N/A	0 (0)
**Evaluation of teachers in clinical teaching**		
Teacher evaluation: Preferred teachers		48 (29)
Large-group teaching	Some teachers are skilled at lecturing eloquently and give us examples of the types of diseases observed in a department (S19).	9 (4)
	A good teacher teaches you how to think and solve problems rather than teaching by the book and never teaching you to think for yourself (S21).	
Small-group teaching	[Teachers] incorporate case studies and detail the disease diagnosis, the cause of onset, clinical symptoms, physical examinations, and disease-specific examinations…diagnostic thinking, subsequent treatment plans for this medical case, the next disease, and a preliminary understanding of the recovery process (S7).	12 (5)
Bedside teaching	[Teachers] lead you around the department to observe patients and inform you about the general diagnosis of a disease. They also talk about treatment plans. They might give you a demonstration or ask you to read books and then allow you to work under their supervision (S13).	37 (25)
	[Teachers] allow you to work on your own. For instance, they allow you to do everything, including holding patient consultations, performing work-ups, and reviewing the literature, and then they explain everything to you later (S14).	
	My senior colleagues really care about us. If they see that we are exhausted from overtime, are planning to leave, or are tired from seeing patients, they ask us to join them for dinner, relax a bit, or sing karaoke. It feels good. If we do not understand certain things in practice, they are always there to help us. If we feel that a patient did not respond well to treatment, they step in and assist us (S12).	
Teacher evaluation: Unpreferred teachers		37 (15)
Large-group teaching	The unpreferred teachers are those who teach in public theory classes [large classroom setting]…some of them appear to be in a hurry. They speak very quickly and do not often interact with us. They do not even know if we understand them in class; they just talk, talk, talk (S15).	9 (5)
Small-group teaching	Communicating [with an unpreferred teacher] is difficult if they teach in a different department or are not my teacher (S11).	1 (1)
Bedside teaching	Sometimes, I feel that we go through training to learn; however, when a teacher dislikes teaching us, I feel frustrated and less passionate about learning (S14).	27 (12)
	Because I do most things, [teachers] sometimes check what I have done. When experiencing difficulties and feeling uncertain about a physical examination, if the teachers do not double-check things, I feel insecure (S23).	
**Reflection on improvements and future learning directions and methods**		103 (29)
Incompetency in the present stage and reasons for this incompetency	Mentally, I am satisfied; however, I lack certain competencies in practice. Because we are very busy at work—I’m not saying that only we are busy, the entire medical system is backed up—we want some time off to take a break. Mentally, we really want to [improve our competencies]; however, our physical performance cannot keep pace with our mental performance and we therefore slack off a bit (S12).	6 (5)
Competencies to be improved at the present stage	My diagnostic skills are still developing. With regard to my communication skills, I am definitely not careful enough when managing patients with serious conditions (S1).	21(16)
	[I] must improve my theoretical knowledge and skills…because I wish to specialize in intensive care (S29).	
Future learning directions	The most important aspect is diagnostic skills…which require theoretical learning and clinical practice. The more you observe, the more confident you are in making a preliminary diagnosis of a disease. For example, our director conducting ward rounds can accurately identify a disease as soon as he observes the [test] results (S12).	27 (15)
	I feel that English is a major weakness of mine. Although I am somewhat proficient [in English], I would like to access more up-to-date information…What I mean by “science and research competency” is not studying various topics but having the ability to obtain up-to-date knowledge (S5).	
Future learning methods	First, I must find a good clinical learning environment. Therefore, I must find a hospital where I can be exposed to numerous cases of various types. I must use this environment as a platform to improve myself. Second, [I must] continually improve my learning [method]…I will examine the relevant forums that I mentioned earlier and read books (S14).	15 (7)
	I will attempt to learn and read more, watch videos, and search for information online…I will search for information online and learn from senior doctors with many years of experience (S13).	
Future career planning	To be honest, at one time, I thought about teaching and not becoming a doctor…[I] had this idea of pursuing a teaching career directly after my residency. In practice, some tasks are not what I imagined and not exactly what I wish to do (S4).	7 (5)
Improvements to education systems	Each department should prepare us before we begin training with them and inform us about the diseases most commonly observed in admitted patients. Because each department has a specialization, especially in the current era of classifying specialties to the smallest detail, as soon as we begin training in a department, we should focus our attention on the commonly observed diseases (S12).	27 (19)

^a^The first number indicates the total number of times the theme or subtheme was mentioned; the number in parentheses represents the number of residents who mentioned the theme or subtheme (residents may mention the same theme or subtheme multiple times)

### Challenges and reflections in the early phase of clinical learning

Although the residency training program is well-designed, residents encounter challenges during clinical learning resulting from a lack of individual competency, prior knowledge, and learning motivation. Residents can face these challenges at the beginning of their residency when they engage directly with their patients and feel responsible for the patients’ welfare. Residents can experience a lack of competency or confidence, communication difficulties, and language barriers and be met with feelings of distrust from patients and the medical team. These problems are the cause of anxiety and unease; however, some residents obtain a sense of accomplishment from curing patients.

In reflecting on their residency, some residents perceive their responsibility to patients as being the most crucial aspect of residency. They do not only learn from teachers; they also learn from addressing the problems they face in clinical practice. Moreover, residents improve their knowledge, skills, and communication techniques by adopting different independent learning methods, such as discussing case studies and reading journal papers.

### Evaluation of teaching methods and designs in clinical medicine

Large-group, small-group, and bedside teaching are the three main teaching methods used in residency training. The study participants’ evaluations of these three teaching methods are described in the following section.

#### Large-group teaching

The participants believed they could acquire more knowledge in large groups than in small groups. However, some of them reported that their motivation to learn in large groups was lower if the teacher lacked teaching skills, failed to consider students’ competency differences, and used materials that were difficult to understand or unrelated to clinical practice.

#### Small-group teaching

Regarding teaching in small classroom settings, several residents stated that they could quickly and systematically acquire practical knowledge from teachers’ class materials. A few of them indicated that small-group teaching facilitated quick and dedicated learning and provided a complete understanding of a patient’s diagnosis and the full treatment process. Immediate feedback from teachers also contributed to effective learning. Some participants reported that the solely theoretical analysis of cases discussed in small-group teaching reduced their learning motivation. Moreover, the content of small-group teaching sessions often overlapped that encountered in clinical training sessions, meaning that attendance of small-group teaching was a mere formality.

#### Bedside teaching

Most of the residents considered bedside teaching advantageous as an opportunity to learn directly from patients. For teaching at bedsides and on ward rounds, teachers typically introduce a patient and then provide diagnostic interpretations and demonstrations of treatments. For each medical case, residents engage in self-learning by combining practice and theory, which improves their diagnostic thinking process. Several residents reported learning from other medical personnel during bedside teaching. For instance, they learned communication skills and medication administration knowledge from nurses and pharmacists.

#### Informal curriculum

In addition to the aforementioned teaching activities, the residents engaged in learning through informal courses. A few mentioned that when the hospital organized a clinical skills competition, it offered additional tutorials and practice exercises for residents, which improved the residents’ learning motivation and performance.

### Evaluation of teachers in clinical teaching

#### Preferred teachers

Several residents indicated that teachers who taught in large groups were preferred over those who taught in small groups if they had strong teaching skills. Combining teaching materials with real-life case studies improves residents’ learning motivation and performance. Some residents believed that teachers of small groups were effective if they explained the entire treatment process for clinical case studies that had arisen during previous residencies and if they incorporated new medical knowledge and discussions on medical development to familiarize residents with new treatment approaches.

Many of the residents indicated a preference for clinical teachers who respected and cared about the residents. One resident described how his sense of belonging to a team increased when a teacher discussed complex patient diagnoses and treatments with residents during a social gathering. In the medical field, preferred teachers both provide a learning experience that transforms students into health care providers and offer assistance and guidance when necessary.

#### Unpreferred teachers

Several residents asserted that, regardless of whether teaching was conducted in a large or small group, they were less motivated when the teacher lacked effective teaching skills or did not consider students’ competency level. A few of the residents stated that they were less motivate when a clinical teacher was not motivated to teach or failed to provide clear corrective instruction when a resident had dealt with a patient ineffectively.

### Reflection on improvements and future learning directions and methods

The residents’ reflections on their clinical training and future learning directions included thoughts about incompetency in the present stage and the reasons for this incompetency, future learning methods, and recommendations for current clinical education systems.

#### Incompetency in the present stage and reasons for this incompetency

The residents asserted that they were presently not fully competent because they had insufficient learning and experience at a medical school. Residents are often too stressed and busy to learn effectively from clinical work and lack an active learning attitude.

#### Future learning directions and methods

Several residents lacked not only knowledge, skills, and diagnostic capability but also the ability to communicate with patients. The residents mentioned that they had to focus on improving their knowledge and skills as well as their patient–physician communication skills. Most of the residents believed that they had to strengthen their ability to communicate with and care for patients. A few residents stated that they had to improve their English proficiency and biomedical research capability to remain informed about the latest medical development trends.

The residents suggested that clinical and active learning should be employed as the main learning methods in the future to develop general medical competencies. The residents wished to strengthen their knowledge base by gaining more clinical experience and asking the senior physicians questions; to improve their clinical skills through learning by doing; and to enhance their patient–physician communication skills by observing how senior physicians engage with patients and manage patients’ conditions in clinical practice. Some residents also wished to undergo clinical learning in other departments. In addition to clinical learning, reading professional books and online learning were reported to be other learning methods that can be adopted by residents.

#### Future career planning

The residents’ goal after their residency was to pass the national examination. However, considering burnout because of their heavy workload and disrespect for physicians from some patients and their family, some residents mentioned the possibility of joining the academic sector, such as by enrolling in graduate school or by becoming a university teacher.

#### Recommendations for current clinical education systems

On the basis of their residency experiences, the residents provided recommendations for medical schools and hospitals. Regarding medical schools, the residents recommended early clinical exposure for medical students to provide a solid basis for medical learning. The residents proposed that hospital administrators adjust the length of training for different departments and prolong residents’ training in the department they were assigned to practice upon completion of their residency. They also suggested that additional tests be organized to increase students’ learning motivation and that language communication and online courses be implemented to enhance the effectiveness of large-group learning. Hospitals can provide a list of the diseases that they commonly encounter to enable residents to learn to treat patients within a limited period of time and quickly assimilate into the departmental environment.

## Discussion

A participant stated, “In a clinical setting, you feel that you are not a student anymore but rather a physician” (S 2). During the participants’ initial period of residency training, they perceived a change in their identity, performed various responsibilities and tasks, and entered a new life stage. Various factors relating to residents’ ecology of education—such as their interactions with patients and patients’ family members, teachers, and medical staff and their participation in multimethod teaching—affected their process of learning and development.

### Challenges in clinical learning

Effective patient–physician communication is a fundamental factor influencing the quality of health care [15]. Effective communication facilitates medical decision-making and increases the degree to which patients comply with physicians’ instructions. Moreover, it increases patient and physician satisfaction with health care outcomes [16]. The present study revealed that in the early stage of hospital residency training, residents face challenges including a lack of confidence in their medical competency and an inability to communicate effectively with patients and patients’ family members. Because some of the residents participating in this study were unfamiliar with local dialects (e.g., S 28), they were unable to simplify medical terminology to clearly and accurately explain a patient’s medical conditions to the patient. We propose that a lack of medical competency and ineffective communication skills cause patients and medical team members to distrust residents, and such distrust diminishes the residents’ confidence in their clinical practice abilities.

The residents believed that they should attend courses to improve their medical competency and patient–physician communication skills in addition to learning through clinical experience (e.g., S 22). They must also continue to learn through bedside teaching, observing the process of communication and treatment between senior physicians and patients. Such observation would strengthen the medical competency of the residents. After the challenges in the early phase of training are addressed, the demanding nature of healthcare workloads and patients’ over involvement in the healthcare process push residents into the burnout stage.

Job burnout is a growing problem among young health care professionals. In China, burnout affects not only the physical and mental health of future doctors but also their empathy for patients, confidence in their professional role, and professionalism [17]. This study determined that burnout in residents is caused by a heavy workload and society’s disrespect for doctors. In residents, burnout can lead to unfavorable learning outcomes and job switching, with some residents potentially enrolling in graduate education and then teaching at a university rather than continuing in clinical practice (e.g., S 4).

### Evaluation of the teaching methods and designs in clinical medicine

#### Large-group teaching

Teaching in large classroom settings is an economical approach for delivering information to large groups of learners [18]. Several residents maintained that lectures can impart a wide range of basic knowledge (e.g., S 27); however, the residents stated that they were not motivated to learn in a large classroom setting if the teacher lacked effective teaching skills, failed to consider students’ competency differences (e.g., S 3), and used materials that were difficult to understand or unrelated to clinical practice (e.g., S 12). This study determined that online teaching, the main purpose of which is to reduce the time spent on passive teaching, can improve learning performance in large classroom environments. This result is consistent with that of a previous study [19]. Flipped classrooms and the incorporation of interactive technology can increase the efficiency of large-group teaching [20]. Nevertheless, a student-centered learning approach remains the most crucial approach to improving learning outcomes in large classroom environments.

#### Small-group teaching

Several residents indicated that small-group learning involves case-based learning, whereby residents apply knowledge in practice to enhance their medical competency and identify solutions to medical problems (e.g., S 18). This finding is in agreement with that in related research [21]. However, high-quality small-group learning experiences are difficult and expensive to create because this process is labor-intensive and time-consuming [22].

#### Bedside teaching

In bedside teaching, residents learn through observation, simulation, and practice [2, 3]. Bedside teaching enables residents to acquire medical knowledge, clinical capabilities, interpersonal communication skills, and professionalism from senior physicians and health professionals [1]. Most of the participants (e.g., S 8 and S 2) in this study believed that the theoretical and clinical application of knowledge and experiential learning in bedside teaching not only enables the resolution of clinical problems but also promotes residents’ self-learning and improves their diagnostic thinking. The role models from whom residents learn are not confined to senior physicians. Residents also learn health care behaviors and knowledge, including medication administration and patient care practices, from health professionals such as pharmacists and nurses (e.g., S 18 and S 29). We argue that bedside teaching is the most effective learning method in residency training programs.

#### Informal curriculum

A few residents (e.g., S 12) reported effective learning outcomes and increased self-confidence when they were directly observed and received feedback from teachers when repeatedly practicing clinical skills during clinical skills competitions. This finding is consistent with that of a relevant study [23].

Residency training programs are mainly based on a type of adult learning called situated learning [24]. Situated learning is not abstract learning but rather emphasizes teaching in a real-life environment in which students’ learning behavior originates from solving a problem [25]. Adult learning is characterized by an immediate learning outcome [26]. In this study, the residents (e.g., S 12, S 22, S 18, and S 2) generally preferred to learn through real-life clinical problems because the knowledge they acquired helped them solve the problems they were currently facing and triggered relevant associations that supported the development of medical competencies.

### Which teachers are preferred?

Teachers must trigger and sustain students’ learning motivation and guide them in achieving their learning objectives [27]. This study revealed that residents (e.g., S 15) negatively reviewed teachers that had low teaching motivation or who were reluctant to interact with their students. By contrast, teachers were considered more effective if they had excellent teaching skills and respected their students. Preferred teachers lecturing in a large classroom setting can apply their strong teaching skills and incorporate real-life examples into lecture materials (e.g., S 19). When teaching in small groups, preferred teachers focus on the entire treatment process in a case report, providing residents with in-depth and complete understanding of a case and motivating them to learn (e.g., S 7). In bedside teaching, teachers were regarded as more effective if they gave the participants balanced direction, enabling the participants to gain hands-on experience practicing and performing clinical skills that enabled them to develop their diagnostic thinking and medical problem-solving skills (e.g., S 14). Moreover, these teachers gave the residents assistance and guidance when necessary, ensured patient safety, and reassured the residents when they performed clinical tasks (e.g., S 12). Residents are often eager to gain the respect and understanding of others while learning. Therefore, preferred teachers encourage students to self-direct learn rather than aim to control what the students think [28].

The relationship between an effective teacher and a resident is similar to the relationship between an effective advising professor and a graduate student. Preferred teachers make learners perceive that they are members of a team [29]. This study indicated that in a professional capacity, preferred teachers encourage questions from residents and answer their questions with additional questions to encourage residents to draw on their experience (e.g., S 13 and S 14). In a personal capacity, preferred teachers care about residents and share their work experiences (e.g., diagnosis and treatment of patients with complex disease conditions) with them. Similar to effective mentors, preferred teachers have a critical influence on residents’ academic success, professional growth, and personal development [30, 31].

### Resident learning and the educational environment

Resident learning occurs in a multilevel ecosystem composed of various environmental factors, including patient–physician communication and relationships, teacher–student relationships, teaching resources, and teaching methods (e.g., small-group discussion, bedside teaching, in-person demonstrations, and chalk talk) [4]. Each environmental factor is characterized by the attributes of the cultural ecology embodying the symbiotic relationship between the people (i.e., patients, teachers, and residents) in a classroom setting and their dependence on and role within the environment. Similar to how the ecological environment affects the growth and survival of biological organisms, the aforementioned environmental factors exert a promotive or suppressive effect on how residents address challenges, learn, and develop. Therefore, we assert that the learning and development process of residents is not unidirectional teaching or learning but instead entails the constant continual recall of and reflection on experience; this idea is grounded in ecology of education theory [6]. Resident training course designs must be based on the specific learning environment and learning system of residents and must provide students with a learning environment that promotes self-actualization and humanistic development [32]. Courses for residents must be designed such that they (a) create a safe, interactive, collaborative, and respectful learning environment and (b) integrate residency training—including task, group, and material learning—with clinical elements, enabling residents to perceive that the learning process positively influences their career development and learning motivation.

This study has some research limitations. First, the participants in this study were recruited from a single location, Qingyuan People’s Hospital. Considering the vast territory of China, the results and research conclusion might not be applicable to residents in other parts of China. Second, this study was also limited by our use of deliberate sampling, although the effect was controlled through theoretical saturation. Third, participant interview was the only method of data collection employed. If other types of data had been collected and analyzed, biases would have been minimalized.

The ecosystem of residency education includes both the commonalities of learning theories and the differences in sociocultural environments. The residents’ preferred teachers and teaching methods identified in this study are the same as those reported by the related research [17, 19, 21, 26–31]. Residents’ preferred teachers and teaching methods do not differ depending on whether the residents live in a Chinese or Western culture. The results of this study also indicate that Chinese culture affects Chinese residents’ learning processes. For example, the burnout of residents may be more likely in the context of a Chinese patient–physician relationship. Residency training must be conducted in an environment that facilitates resident learning and must offer meaningful and valuable learning activities relating to each factor of the resident-specific ecosystem of education.

In this study, we obtained data using a quantitative research method grounded in learning and teaching theories and reached valuable conclusions about the interaction and effects of the residents’ learning processes and personal traits within the residents’ sociocultural context. This study has profound implications for residents’ educational course design and also assists in filling the research gap regarding sociocultural effects on medical education. This study can also serve as a foundation for related research in the future.

In this study, we proposed that the interdependent symbiotic relationship existing in the ecosystem of education can serve as a reference for designing residency programs. Future studies can explore the effects of various factors in the ecology of education on resident learning.

## Supplementary Information

**Additional file 1.** The interview outline.

## Data Availability

The datasets generated and analyzed during the current study are not publicly available due to privacy concerns but are available from the corresponding author on reasonable request.
