# Supplementary material for: Qualitative study of the learning and studying process of resident physicians in China
Source: BMC Med Educ. 2022 Jun 15;22:460. doi: 10.1186/s12909-022-03537-x (PMC9202223; doi:10.1186/s12909-022-03537-x)
Supplement: Supplementary file 1 — Additional file 1. The interview outline. [file 12909_2022_3537_MOESM1_ESM.docx]

**The interview outline**

1. **Clinical learning experiences and reflections on learning**
   1. In the clinical medical education courses you have taken in the past, what are the major teaching methods used by the instructors? What kinds of help did these methods offer to your learning? Or maybe they were not helpful?
   2. In your memory, which clinical medical education course has the teaching methods that you like the most? Which one has the teaching methods that you like the least? Please describe them separately and explain why.
   3. During the process of learning, what kinds of positive learning behaviors have you shown?
   4. How would you evaluate your learning behaviors, attitude and outcome? Do you think you have acquired the knowledge and capabilities that a good doctor need to possess?

**2. Experiences working with medical team members**

2.1. During the residency, what are the more remarkable clinical learning interactional experiences (between you and the instructor or the clinical workers)? How did these interactional experiences benefit your clinical learning?

2.2. During the residency, what are the relationships between you and the instructors and the clinical workers? Are such interactional relationships similar to or dissimilar from your expectations? What might be the causes for any dissimilarities?

2.3. According to your residency experiences, what kinds of difficulties have you encountered clinically (such as anything that happened in the symptom-diagnosis-treatment cycle)? What is the most difficult (such as invasive treatment, reaching a medical diagnosis, explaining the medical condition to the patient and their family)? Why? What factor in the medical training is it related to? What courses or course contents need to developed in medical training to overcome such difficulties?

2.4. According to your residency experiences, what parts of the residency program should be modified? How might the residency program be adjusted to help you receive a better clinical training and therefore achieve better clinical performances?

**3. Experience interacting with patients**

3.1. In your residency, what did you interact with the patient? What parts do you consider to be more difficult?

**4. Future learning direction**

4.1. How could residency program be adjusted or strengthened to achieve the ideal residency education that you expect?

4.2. What kinds of learning do you still need to pursue to become a good doctor?
